# Examining Links Between Distinct Affective States and Tobacco Lapse During a Cessation Attempt Among African Americans: A Cohort Study

**DOI:** 10.1093/abm/kaae020

**Published:** 2024-05-13

**Authors:** Dusti R Jones, Lindsey N Potter, Cho Y Lam, Chelsey R Schlechter, Inbal Nahum-Shani, Christopher Fagundes, David W Wetter

**Affiliations:** Center for Health Outcomes and Population Equity (HOPE), Huntsman Cancer Institute and the University of Utah, Salt Lake City, USA; Center for Health Outcomes and Population Equity (HOPE), Huntsman Cancer Institute and the University of Utah, Salt Lake City, USA; Department of Population Health Sciences, University of Utah, Salt Lake City, USA; Center for Health Outcomes and Population Equity (HOPE), Huntsman Cancer Institute and the University of Utah, Salt Lake City, USA; Department of Population Health Sciences, University of Utah, Salt Lake City, USA; Center for Health Outcomes and Population Equity (HOPE), Huntsman Cancer Institute and the University of Utah, Salt Lake City, USA; Department of Population Health Sciences, University of Utah, Salt Lake City, USA; Institute for Social Research, University of Michigan, Ann Arbor, USA; Center for Methodologies for Adapting and Personalizing Prevention, Treatment, and Recovery Services for SUD and HIV (MAPS Center), University of Michigan, Ann Arbor, USA; Department of Psychological Sciences, Rice University, Houston, USA; Center for Health Outcomes and Population Equity (HOPE), Huntsman Cancer Institute and the University of Utah, Salt Lake City, USA; Department of Population Health Sciences, University of Utah, Salt Lake City, USA

**Keywords:** Tobacco cessation, Positive emotion, Negative emotion, Distinct emotions

## Abstract

**Background:**

Affect states are posited to play a pivotal role in addiction-related processes, including tobacco lapse (i.e., smoking during a quit attempt), and distinct affective states (e.g., joy vs. happiness) may differentially influence lapse likelihood. However, few studies have examined the influence of distinct affective states on tobacco lapse.

**Purpose:**

This study examines the influence of 23 distinct affect states on tobacco lapse among a sample of tobacco users attempting to quit.

**Methods:**

Participants were 220 adults who identified as African American (50% female, ages 18–74). Ecological momentary assessment was used to assess affect and lapse in real-time. Between and within-person associations testing links between distinct affect states and lapse were examined with multilevel modeling for binary outcomes.

**Results:**

After adjusting for previous time’s lapse and for all other positive or negative affect items, results suggested that at the between-person level, joy was associated with lower odds of lapse, and at the within-person level, attentiveness was associated with lower odds of lapse. Results also suggested that at the between-person level, guilt and nervous were associated with higher odds of lapse, and at the within-person level, shame was associated with higher odds of lapse.

**Conclusions:**

The present study uses real-time, real-world data to demonstrate the role of distinct positive and negative affects on momentary tobacco lapse. This work helps elucidate specific affective experiences that facilitate or hinder the ability to abstain from tobacco use during a quit attempt.

## Teaser

Feelings of joy, attentiveness, guilt, shame, and sadness are associated with tobacco lapse among people who identify as African American and are trying to quit.

## Introduction

Although the prevalence of tobacco use has declined precipitously in the last 50 years [1], tobacco continues to be the leading cause of preventable death in the USA [2]. Tobacco use remains a key risk factor for major chronic diseases, including both cancer [3, 4] and coronary artery disease [5]. Although the vast majority of tobacco users indicate they would like to quit (~70%; 6), very few are successful on a given quit attempt. For instance, in 2018, 55% of tobacco users indicated they had made a quit attempt in the past year, yet only 8% successfully quit for at least 6 months [6]. Previous work suggests that most tobacco users (68%) lapse (i.e., any smoking during a quit attempt) within their first week of a quit attempt [7], highlighting the difficulty of maintaining abstinence for even short periods.

Substantial health inequities exist in tobacco use, tobacco-related health outcomes, and tobacco cessation. Despite smoking fewer cigarettes per day [8], people who identify as African American and who smoke have a greater likelihood of experiencing adverse health outcomes [9] compared to those who identify as European American or Hispanic. Although they report a greater desire to quit [10] and make more frequent quit attempts [10], people who identify as African American and smoke have more difficulty quitting than the general population of people who smoke [11]. As such, there is a critical need to understand risk and protective factors influencing lapse during a quit attempt among those who are African American.

Affective states are posited to play a pivotal role in addictive processes more broadly [12–14] and in tobacco lapse [15–17]. Despite the critical need for work elucidating affective precipitants of lapse, few studies have examined distinct affect states (i.e., specific states such as joy—as opposed to more general measures of positive or negative affect), despite theoretical assertions that certain distinct affect states (e.g., shame, guilt, pride) may uniquely influence lapse [18, 19]. As such, the present study examines 23 distinct affect states to help determine which states may be associated with a greater risk for lapse, and which states may protect against lapse in a sample of people who self-identify as African American.

### General Measures of Positive and Negative Affect and Associations with Lapse

Most previous research on the role of affect in tobacco cessation has focused on associations between tobacco lapse and general measures of positive or negative affect. Affect measures encompass multiple distinct affect states, which are often grouped by valence such as the positive and negative affect subscales in the Positive and Negative Affect Scale [20]. Theoretical models in affective science posit that such general positive affect may increase self-regulation in the service of longer-term goals such as tobacco cessation [21, 22] by broadening an individual’s scope of attention, thoughts, and actions in ways that enhance coping resources and promoting engagement in more beneficial health behaviors [21, 22]. General positive affect has been found to be protective against lapse, independent of the effects of general negative affect [16, 23, 24]. Individuals with higher positive affect before a quit attempt reported higher likelihood of abstinence six months postquit [24] and higher momentary positive affect protected against lapse in the first 10 days following a quit attempt [16].

In contrast to the mostly beneficial influence of positive affect on tobacco cessation, general negative affect has a detrimental influence on tobacco cessation. Individuals with higher negative affect had a higher likelihood of lapse [25] and were more likely to lapse earlier during a quit attempt [26]. Similarly, those who had increasing negative affect in advance of their scheduled quit date were less likely to be abstinent at 3-months postquit [27]. Increases in momentary negative affect during a quit attempt have been associated with stronger temptations to smoke [28], and a greater likelihood of lapse that same day [29] and up to 12 h after the initial increase in negative affect [30]. Momentary negative affect has also been found to mediate links between stress and lapse, providing evidence for negative affect as a primary mechanism through which stress may influence lapse [15].

### Distinct Affective States and Lapse

In addition to general measures of positive and negative affect, distinct affect states are consequential for processes including health-relevant decision-making and tobacco cessation [31]. Indeed, even the ability to differentiate and identify distinct affect states is important for tobacco cessation processes [32–34] as this is a crucial skill for self-regulation [35]. Not only do distinct positive (e.g., happiness, gratitude, pride) and negative affect states (e.g., anger, sadness, shame) likely have differential impacts on cognition and behavior [36, 37], but examining distinct affect among tobacco users who identify as African American is critically important given previous research suggesting that distinct affect states may carry different risk or protective effects for African Americans than European Americans [38].

#### Distinct positive affect states

Although we know of only one study that examines distinct positive affect states on tobacco lapse [39], social and motivational models [40, 41] suggest that several distinct positive affect states, such as pride and happiness, may differentially influence goals, behavior change, and health [42, 43]. Some positive affect states, such as pride and gratitude, likely influence tobacco cessation through social processes. Pride generally occurs when individuals feel success or achievement related to status or goal-attainment [44] and is closely associated with motivation when a goal or event is linked to one’s identity [45]. Pride has been found to predict engagement in more beneficial health behaviors [46], particularly when behaviors, such as tobacco cessation, are socially desirable. Gratitude, an affective state of thankfulness for the benefits that one receives from others [47], may also promote goal-oriented behaviors by increasing motivation to circumvent short-term rewards (e.g., alleviation of tobacco craving) in favor of longer-term goals (e.g., good health, tobacco cessation [40, 48];. Gratitude tends to promote behaviors via pro-social processes when, for example, individuals feel that cessation would benefit their social group (e.g., protecting family from secondhand smoke).

Other positive affect states, such as feelings of joy, happiness, enthusiasm, and relaxation promote engagement in achievement-oriented behaviors [21], which may help with tobacco cessation. Previous work indicates that higher daily happiness, enthusiasm, and relaxation are associated with slower time to first lapse [39]. Work in other health behaviors suggests that associations between positive affect and health behaviors vary by the arousal-level of the affect item, such that more activating positive affect states (e.g., joy) demonstrate stronger associations with health behaviors compared to more deactivating positive affect states (e.g., feeling relaxed) [49–52]. This may be because more activating positive affect states tend to be approach-oriented, meaning that they motivate active engagement in behaviors [53]. In practice, this may result in engagement in behaviors that facilitate successful tobacco cessation, such as putting on nicotine patches to mitigate cravings. In contrast, feelings of relaxation and other deactivated positive affect states are considered to be more restorative [50], and may not motivate approach-oriented behaviors to the same extent as activating positive affect states, resulting a weaker effect on tobacco lapse. Thus, we hypothesize that joy, enthusiasm, happiness, and relaxation will be associated with lower lapse likelihood, but that associations will be stronger among the more activating states (e.g., joy, enthusiasm).

#### Distinct negative affect states

Compared to the limited work linking distinct positive affect states with tobacco lapse, more work has examined distinct negative affect states and their associations with lapse. Several studies have focused on anger, sadness, restlessness, shame, and guilt. Although anger is also an approach-oriented emotion [53], it has been associated with greater risk-taking and decreased perceptions of risk for future health problems [54], and with difficulty quitting tobacco [55, 56]. Thus, although anger is an approach-oriented state, feelings of anger may lead to behavioral engagement in maladaptive behaviors that increase the risk of tobacco lapse. Moreover, nicotine seems to decrease anger [57], reinforcing tobacco use as a way to successfully regulate anger.

Similar to anger, sadness, and restlessness increase efforts to immediately alleviate such feelings, leading to a greater likelihood of lapse. Sadness and restlessness are common symptoms of withdrawal that trigger increases in cravings and subsequent lapses [58]. For instance, restlessness, or the feeling of being jittery, has been related to increased craving for nicotine as nicotine reduces restlessness [28, 59–61]. Interestingly, sadness increases sensitivity to short-term rewards [62] and has been associated with the urge to smoke in studies using EMA [56]. Thus, sadness may be reinforcing for tobacco use as the feelings of relief and reward when smoking after feeling sad may be especially strong. Supporting this premise, sadness seems to have a lasting influence on tobacco use, as sadness has been associated with greater likelihood of tobacco relapse even 20 years after a successful quit attempt [19].

Finally, self-conscious affect states such as shame and guilt may also be negatively related to cessation. According to appraisal models [63, 64], shame is associated with global negative feeling towards the self and may result in external attributions (e.g., blaming others or situations) for one’s difficulties and behaviors [18], which may limit feelings of responsibility for maintaining abstinence. Indeed, people who report higher levels of shame also have less likelihood of successful tobacco cessation [65]. Guilt is thought to be more behavior-specific (i.e., feeling bad about a behavior one engaged in, such as lapsing during a quit attempt) and to motivate individuals to resolve said behaviors or circumstances that lead to guilt [18]. However, limited research on guilt and tobacco use has found no association of guilt with cessation or progression from first lapse to relapse, but guilt plays a prominent role in some models of relapse, warranting additional work [66, 67].

### The Present Study: Aims and Hypotheses

Based on extant literature and theoretical work, we hypothesized that at the between and within-person levels, joy, happiness, enthusiasm, relaxed, gratitude, and pride would be associated with lower lapse likelihood, and that more activating positive affect states, such as joy and enthusiasm, would be more strongly protective against lapse than less activating positive affect states (e.g., relaxed). We also hypothesized that anger, sadness, restlessness, shame, and guilt would be associated with higher lapse likelihood. Because very few studies have examined distinct positive and negative affect states, especially among those who identify as African American, we examined the associations of several other distinct positive (active, calm, determined, and attentive) and negative affect states (disgusted, irritable, lonely, nervous, tired, hopeless, scared, and bored) with lapse on an exploratory basis.

Given the importance of nicotine, craving, and urge to use in linking certain distinct emotions (e.g., anger, restlessness) to tobacco lapse, it is likely that the extent to which distinct affective states may be helpful or harmful in maintaining tobacco cessation may depend on how addicted the individual is to nicotine. Thus, on an exploratory basis, we examined whether nicotine dependence moderated the associations between distinct affective states and lapse.

## Methods

This study was approved by the Institutional Review Board at the University of Utah in accordance with the ethical standards laid down by the 1964 Declaration of Helsinki and its later amendments. Data from Break Free II were used; these data were collected between 2017 and 2019. The goal of the study was to examine the influence of intrapersonal and contextual factors on cigarette lapse and abstinence among people who identify as African American or Black and who are current smokers. Participants were from Houston, TX, and outlying areas. Inclusion criteria included being over 18 years of age; smoking at least three cigarettes per day; having an expired carbon monoxide [CO] of six parts per million [ppm] or higher; having a functioning telephone number; having at least a marginal level of health literacy in English; and being motivated to quit using tobacco within the next 30 days. Exclusion criteria included having any known contraindication for the nicotine patch (e.g., heart attack, angina, cardiac arrhythmia, uncontrolled hypertension, skin allergies, or chronic skin disease); being enrolled in a tobacco cessation program within the last 90 days; having another household member enrolled in the study; currently using tobacco cessation medications; and being pregnant or lactating.

### Participant Characteristics

Three hundred two participants enrolled in the study. Of those enrolled, 262 participated in the EMA portion of the study, but 28 did not provide enough EMAs during the postquit period for us to use their data. Additionally, 14 participants were missing data on important covariates. Thus, the final analytic sample was *N*=220. There were no significant differences in demographics (e.g., gender, income, age) or nicotine dependence between those who were retained in the study versus those who were not.

All participants identified as African American. Participants were 50% female, aged 18–78 (*M*=49.10, *SD*=12.26), and reported smoking between 3 and 48 cigarettes per day (*M* = 14.40, *SD* = 7.54). Most participants were single (72%), did not have a college education (62%), and reported household incomes of less than $25,000 per year (67%). Participants responded to 2-3 random EMAs per day on average (~65% compliance).

### Study Procedures

Interested participants were screened for eligibility over the phone. Potentially eligible participants were then invited to attend an in-person information session where they were provided with detailed information about the study and final eligibility was determined. Eligible participants were then scheduled for a baseline visit where informed consent was obtained from all individuals included in the study. Formally enrolled participants attended five in-person visits, including a baseline visit, a visit on their quit date, and three additional visits during the postquit period. Only data from the baseline visit was used in the present study; other visits are not further discussed. At the baseline visit, participants provided demographic information and responses to other psychosocial variables known to be related to tobacco use and were provided with tobacco cessation treatment (see “Treatment” section below).

At their baseline visit, participants were provided with preprogrammed smartphones and trained in following an Ecological Momentary Assessment (EMA) protocol, which included carrying the smartphones for the first 10 days of their quit attempt. Each EMA study day was divided into four intervals that began when participants clicked a button to start their EMA day on the phone. EMAs were designed to be delivered at semi-random times (i.e., one EMA delivered randomly within a 4-h time block) within each of the four intervals with several constraints: EMAs were scheduled such that they must be at least 5 min apart, participants could choose up to 120 min of privacy mode per day where no EMAs would be delivered, and no EMAs would be delivered if the smartphone detected driving.

Financial compensation was given in the form of gift cards and was contingent on participation. Specifically, participants received between $30 and 80 for completing each in-person visit. Participants were also eligible to receive compensation for completing EMAs and wearing study equipment (i.e., cardiovascular monitors—data not used in the present study, $1.25 for each completed EMA if they wore the study equipment for at least 60% of the time since the previous EMA, or $0.50 for each completed EMA if they were not wearing the study equipment for at least 60% of the time since the previous EMA). In total, participants were eligible to receive up to $450 in compensation for completing the entire study protocol.

### Treatment

At each lab visit, all enrolled participants received nicotine patch therapy (for a total of 6 weeks of therapy), self-help materials, and brief quitting advice based on the Treating Tobacco Use and Dependence: Clinical Practice Guideline [68]. These materials are designed to provide information on identifying and planning for high-risk situations, coping with tobacco urges, and planning for a nonsmoking lifestyle.

### Measures

#### Positive and negative affect

Each EMA survey asked participants to indicate how much each affect-related word described them at that moment on a scale from 1 (strongly disagree) to 5 (strongly agree). Positive affect states were assessed with 10 items (active, calm, determined, enthusiastic, grateful, proud, happy, joyful, attentive, and relaxed) and negative affect states were assessed with 13 items (angry, shame, disgusted, guilty, irritable, lonely, nervous, sad, restless, tired, hopeless, scared, bored).

#### Lapse

In each EMA, participants were asked, “Since the last assessment, have you smoked any cigarettes?” This single-item cigarette lapse variable was indicated by a yes/no response (dummy coded: 0 = no, 1 = yes).

#### Demographic characteristics and covariates

Covariates were measured during the baseline assessment and included gender, partner status, income, and age. Additionally, the number of cigarettes participants typically smoked per day was measured at baseline as an indicator of nicotine dependence. Gender (0 = female, 1 = male), partner status (0 = single, 1 = married/cohabiting), and income (0 = household income below $20k USD per year, 1 = household income $20k-<40K per year, 2=$40k+ per year) were effects coded.

### Analytic Plan

The present study examined the influence of each affective state on lapse during the postquit period. We separated the between from the within-person variance by group-mean centering for between-person effects and person-mean centering for within-person effects. We lagged each affect item so that for all models, affect at time *t* would predict lapse during the interval between *t* and *t* + 1. All data manipulation and analyses were conducted using SAS 9.4. We used multilevel models to test all hypotheses. Lapse was regressed on each individual affect item at the between (Level 2) and within person levels (Level 1) via PROC GLIMMIX with a logistic link function and outputting all results as odds ratios. All models included gender, age, income, partner status, and nicotine dependence as covariates. Random intercepts and an AR(1) covariance structure were included in all models; random slopes were tested and included if significant at *p* < .05. Finally, to test exploratory interactions between nicotine dependence and distinct affect on lapse, interaction terms between nicotine dependence and each affect state were added to models described above. Interaction terms were modeled at between and within-person levels. Significant interaction terms were probed with simple slopes at ±1*SD* for nicotine dependence. Because hypotheses were specified *a priori* and because this is one of the first studies to examine the effects of distinct positive and negative affect on lapse, we do not correct for multiple tests.

## Results

### Preliminary Analyses

None of the covariates were significantly associated with tobacco lapse (gender: *OR* = 0.73, *CI:* 0.40, 1.31; partner status: *OR* = 0.77, *CI:* 0.41, 1.44; income: *OR* = 1.43, *CI:* 0.97, 2.10; age: *OR* = 1.00, *CI:* 0.98, 1.03; nicotine dependence: *OR* = 1.03, *CI:* 0.99, 1.08). Nonetheless, given their importance for tobacco lapse in previous work, these covariates were maintained in analyses. Correlations among affective states can be found in Supplementary Tables S1 and S2.

Missingness was not associated with any demographic indicators (i.e., gender, partner status, income, and age) or with nicotine dependence. We also examined whether affective states and lapse were associated with data missingness at the next assessment. Results suggested that feeling more scared was associated with lower odds of missingness at the next assessment (*OR*=0.70, *CI:* 0.56, 0.88), whereas feeling more joyful (*OR*=1.25, *CI:* 1.05, 1.48), more guilty (*OR*=1.31, *CI:* 1.11, 1.55), more hopeless (*OR*=1.17, *CI:* 1.001, 1.39), and having a tobacco lapse (*OR*=1.92, *CI:* 1.55, 2.37) were all predictive of greater odds of missingness at the next assessment. To help address this issue, using the same analytic strategy highlighted in the Analysis Plan, sensitivity analyses were conducted when (i) adjusting for all individual positive or negative affect items, (ii) when adding in a composite measure of positive or negative affect, and (iii) adjusting for lapse at *t* − 1.

All results for positive affect states predicting lapse can be found in Table 1 and all results for negative affect states predicting lapse can be found in Table 2. Below, we only highlight those results that were significant.

**Table 1.** Positive Affective States at Time *t* Predicting Lapse During the Interval Between Time *t* and *t* + 1.

	Intercept			Between-person effects			Within-person effects		
	OR	CI		OR	CI		OR	CI	
Active	1.43	(0.86,	2.38)	0.99	(0.87,	1.13)	0.91	(0.80,	1.04)
Determined	1.44	(0.86,	2.40)	1.11	(0.97,	1.28)	0.89	(0.77,	1.02)
Enthusiastic	1.43	(0.86,	2.38)	1.02	(0.89,	1.16)	1.05	(0.88,	1.24)
Proud	1.42	(0.85,	2.35)	0.98	(0.84,	1.13)	0.94	(0.82,	1.08)
Happy	1.42	(0.86,	2.36)	0.96	(0.83,	1.10)	0.96	(0.80,	1.14)
Joyful	1.39	(0.84,	2.32)	**0.87**	**(0.77,**	0.99)*	0.93	(0.79,	1.11)
Attentive	1.43	(0.86,	2.39)	1.00	(0.87,	1.15)	**0.82**	**(0.71,**	0.95)*
Calm	1.44	(0.87,	2.39)	1.04	(0.90,	1.20)	0.95	(0.81,	1.12)
Grateful	1.44	(0.87,	2.40)	1.12	(0.95,	1.33)	1.01	(0.85,	1.21)
Relaxed	1.40	(0.84,	2.33)	0.92	(0.80,	1.06)	0.97	(0.83,	1.13)

*Notes.* *p < 0.05. Separate models were run for each variable to determine the between and within person association between each affective state and lapse. Covariates were gender, partner status, income, and nicotine dependence.

**Table 2.** Negative Affective States at Time *t* Predicting Lapse During the Interval Between Time *t* and *t* + 1.

	Intercept			Between-person effects			Within-person effects		
	OR	CI		OR	CI		OR	CI	
Angry	1.43	(0.86,	2.37)	1.13	(0.96,	1.33)	1.01	(0.84,	1.21)
Disgusted	1.42	(0.86,	2.35)	**1.22**	**(1.05,**	1.42) *	1.02	(0.89,	1.16)
Irritable	1.42	(0.86,	2.36)	1.07	(0.94,	1.21)	1.00	(0.87,	1.14)
Nervous	1.40	(0.84,	2.33)	**1.23**	**(1.05,**	1.43) *	0.92	(0.78,	1.08)
Scared	1.43	(0.86,	2.38)	1.09	(0.87,	1.37)	1.12	(0.87,	1.43)
Shame	1.43	(0.86,	2.37)	1.18	(0.99,	1.40)	**1.23**	**(1.04,**	1.45)*
Guilty	1.43	(0.86,	2.38)	**1.40**	**(1.16,**	1.69) *	1.08	(0.92,	1.28)
Lonely	1.43	(0.86,	2.36)	1.02	(0.89,	1.18)	0.99	(0.84,	1.16)
Sad	1.40	(0.84,	2.31)	**1.18**	**(1.02,**	1.36) *	0.99	(0.84,	1.16)
Restless	1.43	(0.85,	2.38)	1.03	(0.91,	1.17)	0.98	(0.85,	1.13)
Tired	1.42	(0.85,	2.36)	1.09	(0.97,	1.22)	1.09	(0.97,	1.24)
Hopeless	1.43	(0.86,	2.39)	0.99	(0.84,	1.16)	1.04	(0.86,	1.25)
Bored	1.42	(0.85,	2.37)	1.02	(0.89,	1.17)	1.03	(0.89,	1.18)

*Notes.* *p < 0.05. Separate models were run for each variable to determine the between and within person association between each affective state and lapse. Covariates were gender, partner status, income, and nicotine dependence.

### Aim 1: Distinct Positive Affect States and Lapse

We hypothesized that feeling joyful, happy, enthusiastic, relaxed, grateful, and proud would all be associated with lower lapse likelihood in-the-moment. All other positive affect items were examined on an exploratory basis.

#### Joyful

There was a significant association between feeling joyful and lapse at the between-person level (*OR* = 0.87, *CI:* 0.77, 0.99), such that individuals with a one-unit increase in joy had a 13% lower odds of lapse.

#### Attentive

There was a significant association between attentiveness and lapse at the within-person level (*OR* = 0.82, *CI:* 0.71, 0.95), such that a one unit increase above a person’s own mean in attentiveness was associated with an 18% lower odds of lapse.

#### Sensitivity analysis

We ran sensitivity analyses to determine whether results would hold when all positive affect items were entered into the model. Results were consistent, suggesting that between-person joyfulness (*OR* = 0.84, *CI:* 0.72, 0.98) and within-person attentiveness (*OR* = 0.82, *CI:* 0.70, 0.96) were uniquely associated with lower odds of lapse. Additionally, we examined whether results would hold when a composite positive affect measure was entered into the model. Results were maintained, again suggesting that between-person joyfulness (*OR* = 0.79, *CI:* 0.68, 0.93) and within-person attentiveness (*OR* = 0.82, *CI:* 0.71, 0.94) were uniquely associated with lower odds of lapse. Finally, when adjusting for lapse at the previous time, between-person joyfulness (*OR* = 0.86, *CI:* 0.77, 0.98) and within-person attentiveness (*OR* = 0.85, *CI:* 0.73, 0.98) were uniquely associated with lower odds of lapse. Additionally, however, feeling determined also emerged as a significant predictor of lapse at the within-person level (*OR* = 0.86, *CI:* 0.75, 0.998), such that a one unit increase above a person’s own mean in determination was associated with an 14% lower odds of lapse.

### Aim 2: Distinct Negative Affect States and Lapse

We hypothesized that anger, sadness, restlessness, shame, and guilt would be associated with higher lapse likelihood in-the-moment. All other negative affect states were examined on an exploratory basis.

#### Disgusted

There was a significant association between disgust and lapse at the between-person level (*OR* = 1.22, *CI:* 1.05, 1.42), such that individuals with a one-unit increase in feelings of disgust had a 22% higher odds of lapse.

#### Nervous

There was a significant association between feeling nervous and lapse at the between-person level (*OR* = 1.23, *CI:* 1.05, 1.43), such that individuals with a one-unit increase in feeling nervous had a 23% higher odds of lapse.

#### Shame

There was a significant association between shame and lapse at the within-person level (*OR* = 1.23, *CI:* 1.04, 1.45), such that a one unit increase above a person’s own mean in shame was associated with a 23% higher odds of lapse.

#### Guilty

There was a significant association between guilt and lapse at the between-person level (*OR* = 1.40, *CI:* 1.16, 1.69), such that individuals with a one-unit increase in guilt had a 40% higher odds of lapse.

#### Sad

There was a significant association between sadness and lapse at the between-person level (*OR* = 1.18, *CI:* 1.02, 1.36), such that a one-unit increase in sadness was associated with an 18% higher odds of lapse.

#### Sensitivity analysis

We ran sensitivity analyses to determine whether results would hold when all negative affect items were entered into the model. Results were consistent for feeling nervous, guilty, and ashamed, suggesting that between-person nervousness (*OR* = 1.19, *CI:* 1.002, 1.41), within-person shame (*OR* = 1.22, *CI:* 1.02, 1.47), and between-person guilt (*OR* = 1.40, *CI:* 1.13, 1.73) were all associated with higher odds of lapse. Results for between-person feelings of disgust (*OR* = 1.11, *CI:* 0.93, 1.32) and sadness (*OR* = 1.13, *CI:* 0.96, 1.33) were attenuated to nonsignificance. Additionally, we examined whether results would hold when a composite negative affect measure was entered into the model. Results were consistent with the above suggesting that between-person nervousness (*OR* = 1.18, *CI:* 1.01, 1.39), within-person shame (*OR* = 1.22, *CI:* 1.03, 1.44), and between-person guilt (*OR* = 1.42, *CI:* 1.17, 1.73) were all associated with higher odds of lapse. Results for between-person feelings of disgust (*OR* = 1.19, *CI:* 1.00, 1.42) and sadness (*OR* = 1.12, *CI:* 0.94, 1.32) were again attenuated to nonsignificance. Finally, when adjusting for previous time’s lapse, results were consistent with the main analyses. Between-person nervousness (*OR* = 1.21, *CI:* 1.05, 1.41), guilt (*OR* = 1.38, *CI:* 1.16, 1.66), disgust (*OR* = 1.22, *CI:* 1.06, 1.41), and sadness (*OR* = 1.18, *CI:* 1.02, 1.36), and within-person shame (*OR* = 1.21, *CI:* 1.02, 1.42) were all associated with higher odds of lapse.

### Exploratory Aim: Interactions between Nicotine Dependence and Distinct Affect States on Lapse

On an exploratory basis, we examined the interactions between nicotine dependence and distinct affect states on lapse.

#### Determination

There was a significant interaction between nicotine dependence and feelings of determination at the between-person level (*OR* = 1.02, *CI:* 1.002, 1.04). Simple slopes indicated that for those with less nicotine dependence, higher average feelings of determination were not significantly associated with lapse (*OR* = 0.89, *CI:* 0.77, 1.03). For those with more nicotine dependence, higher average feelings of determination were predictive of greater lapse risk (*OR* = 1.19, *CI:* 1.04, 1.36), such that those with a one-united higher level of determination had a 19% higher odds of lapse.

#### Restlessness

There was a significant interaction between nicotine dependence and feelings of restlessness at the within-person level (*OR* = 1.01, *CI:* 1.002, 1.03). Simple slopes indicated that for those with lower nicotine dependence, higher than typical feelings of restlessness were not significantly associated with lapse (*OR* = 0.91, *CI:* 0.81, 1.02). For those with more nicotine dependence, higher than typical feelings of restlessness were predictive of greater lapse risk (*OR* = 1.17, *CI:* 1.04, 1.32), such that moments where individuals had a one-unit increase in feelings of restlessness had a 17% higher odds of lapse.

## Discussion

Affective states are primary drivers of addictive processes [12]. Modern affective science highlights the importance of not only the ability to distinguish between affective states [32–35] but on the unique influence of distinct affect states on cognitions and behaviors [69, 70]. We hypothesized that feelings of joy, happiness, enthusiasm, relaxation, gratitude, and pride would be associated with lower lapse likelihood, with somewhat stronger associations between higher activation positive affect states. We also hypothesized anger, sadness, restlessness, shame, and guilt would be associated with higher lapse likelihood. Our hypotheses were partially supported. The positive affect states of joy and attentiveness and the negative affect states of nervousness, guilt, and shame uniquely influenced lapse in this sample. Additional analyses suggested that nicotine dependence moderated the effects of determination and restless on lapse, such that these states were predictive of greater odds of lapse only among those with higher dependence. Although there have been some notable efforts related to studying distinct states and tobacco lapse [39, 55, 56], to the best of our knowledge this study provides the most comprehensive assessment of the role of distinct affect states in lapse to date. The results of this study help to identify those affect states that may uniquely influence risk and resilience for lapse among those who identify as African American. Together these findings demonstrate that, at least in this sample, the influence of positive affective states is not always beneficial and given null results, not all negative affect states detrimental to lapse.

Limited previous research has indicated that happiness, enthusiasm, and relaxation, as well as anger, sadness, restlessness, and shame are associated with tobacco use outcomes [39, 59, 60, 71]. Theoretical assertions suggested that a wider variety of distinct affect states should be uniquely associated with lapse [42, 64, 66], yet we found evidence for relatively few associations, particularly in sensitivity analyses. There are some possible reasons for discrepant findings with previous research and our hypotheses. First, although our sensitivity analyses were important to help account for missingness in the data, several affect states were multicollinear at the between-person level, and this could account for null associations with sadness and disgust in sensitivity analyses. Second, the present study specifically examined distinct affect states among those who identify as African American who live in a particular region of the USA. It is possible that there are sample differences that do not generalize to other groups. Although replication is needed with even more comprehensive measures of distinct affective states than examined here, the present study did assess a broad array of affective states. Future research would benefit from further homing in on which states are linked with tobacco lapse, as this would allow us to optimize treatments and interventions designed to target specific affective states in tobacco cessation.

In the present study joyfulness, attentiveness, and determination (in exploratory and supplementary analyses) demonstrated an association with lapse. We had expected that joyfulness, along with other activating positive affect states, would be more strongly associated with lapse based on theoretical assertions that activating states are often approach-oriented [53] and based on previous work demonstrating stronger associations with health behaviors based on arousal [49–52]. Although joyfulness is one of the most activating states in our study, other activating positive states (i.e., enthusiasm) were not associated with lapse. Thus, joyfulness may have unique properties that protect from lapse beyond approach-orientation. It is possible that trait level joy has a stronger association with goal-attainment, motivation, or self-efficacy than the other activating affect states studied here, or perhaps that joy more consistently leads to engaging in approach behaviors that are protective against lapse (e.g., joy always or most of the time leads to individuals putting on a nicotine patch, whereas enthusiasm may lead one to engage in approach behaviors somewhat less often). Our findings also suggested attentiveness and determination were implicated in lapse. Attentiveness is a cognitively oriented state that is implicated in affective processes. According to the Broaden and Build theory, attentiveness increases with increases in other positive affect states [22], yet our sensitivity analyses suggest that within-person, or event-level increases in attentiveness have a unique influence on lapse. Interestingly, for those with high nicotine dependence, determination was associated with a greater likelihood of lapse. Determination is generally associated with high motivation and willingness to maintain goal-oriented behaviors despite challenging circumstances [72]. Although speculative, it is possible that those who have higher trait-level determination but also have stronger addiction to nicotine underestimate the difficulty they will have with the withdrawal process, perhaps over-relying on willpower or grit and underestimating the need for structural support such as nicotine patches [75].

Results for negative affect states suggested that guilt, shame, nervousness, and for those with higher nicotine dependence, restlessness, were all associated with greater lapse likelihood. Findings for guilt and shame are consistent with theoretical models of relapse, particularly with the abstinence violation effect [66, 67, 73], which posits that lapse predicts increases in guilt and shame that, in turn, increase risk for subsequent lapse. Although our results cannot imply causal associations, together with previous work, these findings suggest that a renewed look at how interventions could best reduce trait-level feelings of guilt and event-level feelings of shame, or break the linkage between those affective states and lapse, is warranted. Feelings of restlessness and nervousness have previously been related to increased physiological arousal that is typical of nicotine withdrawal symptoms in tobacco cessation [74]. Consistent with this previous work, results for restlessness were only significant among those with higher nicotine withdrawal and only at the within-person level, suggesting an event-level effect that is related to withdrawal symptoms. Increased feelings of restlessness may be an important forewarning of lapse that could be treated with nicotine (e.g., chewing nicotine gum). In contrast, trait-level associations were evidenced for nervousness, and these effects were not moderated by nicotine dependence, suggesting it is not just a withdrawal process but rather something about those who are higher in trait-level nervousness that heightens the risk for tobacco lapse. A large body of work suggests that individuals higher in nervousness as an indicator of anxiety may use tobacco as a coping mechanism to deal with stress and general feelings of negative affect [75–78]; thus, the loss of a useful coping mechanism, combined with physiological withdrawal, may place these individuals at an increased risk of lapse. Interventions to promote additional coping mechanisms, such as those described below, may be helpful for aiding in tobacco cessation, especially when employed in advance of a quit attempt to help mitigate issues with withdrawal.

Interestingly, the effect sizes for most affected states on lapse were fairly similar, even though some associations were at the between-person level and others at the within-person level. The results highlight the importance of examining both between (trait level—indicating something about the person) and within-person (event-level, indicating something about the situation, environment, context, etc.) effects on lapse. Although between and within-person effects can each be significant (i.e., joy could predict lapse at both the between and within-person levels), we did not see evidence of that here. Rather, our results suggested that significant associations tended to exist at either the between or the within-person levels. In the present study, our results suggested that individuals with more joy, nervousness, and guilt all seem to have trait-level effects on lapse but that the effects of attentiveness and shame on lapse may be more related to the event-level. Although seemingly small, the effects of affect states on moments of lapse risk may accumulate over time, which could have a profound influence on overall tobacco cessation (e.g., an 18% increase in protection against lapse for a one-unit increase in attention in one specific moment may be magnified across multiple moments within a day, across days and weeks). Guilt had a larger effect on lapse compared to other affected states with a one-unit increase being associated with a 40% higher momentary risk of lapse, highlighting the importance of guilt for tobacco lapse.

In light of the present findings, intervention studies might focus on the discrete affect states highlighted here in efforts to determine whether increasing joy and attentiveness and decreasing nervousness, guilt, and shame improve tobacco cessation outcomes among tobacco users who identify as African American. Virtually all behavioral interventions target the reduction of general negative affect and stress to reduce risk, but few specifically target the enhancement of positive affect to build resilience. Previous work has suggested that mindfulness meditation is effective in promoting a broad array of positive affect states and helping to mitigate negative affect states, including nervousness, guilt, and shame [79–83]. Although previous work on mindfulness-based approaches to tobacco cessation has yielded somewhat mixed results [84–86], meditation may address distinct affect states as a proximal mechanism that promotes long-term abstinence. For example, specific aspects of mindfulness may influence joy and attention. Several studies have suggested that meditation exercises, such as acceptance, decentering, and practicing attentional control, increase feelings of joy and attentiveness [87–91]. These positive affect states should, in turn, improve tobacco cessation among those who identify as African American, decreasing abstinence violation effects [86].

Another potentially promising intervention to influence discrete affective states is cognitive behavioral therapy (CBT). Previous work has demonstrated that CBT exercises, such as savoring and cognitive defusion, help mitigate negative affect and promote positive affect [87, 92, 93]. Regarding distinct affect states, CBT has been found to be effective for reducing guilt among those with post-traumatic stress disorder or who have experienced severe stressors [94, 95], and for reducing anxiety and nervousness [96]. Savoring, defined as attending to and appreciating positive affect, helps to prolong enjoyment [87] and may be useful in mitigating urges to use addictive substances such as tobacco [92]. Together, mindfulness interventions and CBT hold promising opportunities to influence the distinct affect states that were found to uniquely relate to tobacco use in this study. However, one important caveat to this premise is that it is relatively unclear whether interventions provide lasting changes in affect, which may be needed to address the between-person effects of affect on lapse. It may be that interventions like mindfulness mediation and CBT need to begin well before participants make an attempt to quit to ensure these have time to influence between-person changes in affect.

### Limitations

The present study has several limitations. Our sample consisted entirely of individuals who identify as African American and are motivated to quit tobacco. Similarly, all participants indicated they were motivated to quit smoking. Thus, our findings that some discrete affective states influence tobacco use may not generalize to populations different than those examined here. Although we examined a broad array of positive and negative affect states, other discrete states may more substantially influence tobacco lapse and cessation efforts. For instance, future-focused positive affect states (e.g., hope) were not assessed in this study but may have a profound influence on tobacco cessation attempts in that these may promote engagement in goal-oriented behaviors. Different types of data may provide additional information on the nature of the connection between distinct affect states and tobacco lapse. Data on the frequency of affective states may provide novel information about which states occur often enough to be an issue for lapse. Qualitative data on connections between affective states and lapse would add a richer contextual understanding that is not possible with the present data. For instance, we do not know whether guilt or shame are directly related to tobacco use (e.g., a participant feels shame because they are craving a cigarette) or indirectly related to tobacco use (e.g., a participant feels shame because they have performed poorly at work). A deeper understanding of what is prompting an affective state from qualitative studies would help us to better contextualize when and how these states may be related to tobacco use.

### Conclusion

This study provides a nuanced look at a wide array of discrete affective states on tobacco lapse among individuals who identify as African American, who use tobacco and are making a quit attempt. It is among the first to make a comprehensive attempt to address multiple discrete affective states in real-world, real-time settings. Our results suggested that joy and attentiveness are protective against lapse likelihood and that nervousness, guilt, and shame are risk factors for lapse. Determination and restlessness also emerged as risk factors for lapse, but only among those with higher nicotine dependence. Although more work is needed to replicate and extend the findings observed here, the present study moves the field forward by homing in on which affective states are most likely to influence tobacco lapse during a quit attempt among those who identify as African American. Future research should examine intervention-induced changes in distinct affect states as proximal outcomes to facilitate tobacco cessation.

## Supplementary Material

kaae020_suppl_Supplementary_Tables_1-2

## Supplementary Data

(1) This study was not pre-registered.
(2) The analytic plan is not registered.
(3) The datasets presented in this article are not readily available because Date/time data cannot be shared given potential to identify individuals with these data. Requests to access the datasets should be directed to david.wetter@hci.utah.edu.
(4) Analytic code are available on OSF: DOI 10.17605/OSF.IO/VMPEB.
(5) Materials are available on OSF: DOI 10.17605/OSF.IO/VMPEB.
