# Supplementary material for: Examining Links Between Distinct Affective States and Tobacco Lapse During a Cessation Attempt Among African Americans: A Cohort Study
Source: Ann Behav Med. 2024 May 13;58(7):506–16. doi: 10.1093/abm/kaae020 (PMC11185091; doi:10.1093/abm/kaae020)
Supplement: kaae020_suppl_Supplementary_Tables_1-2 [file kaae020_suppl_supplementary_tables_1-2.docx]

Supplementary Table 1. Between-person correlations among all affective states.

|  | **Active** | **Determined** | **Enthusiastic** | **Proud** | **Happy** | **Joyful** | **Attentive** | **Calm** | **Grateful** | **Relaxed** | **Angry** | **Disgusted** | **Irritable** | **Nervous** | **Scared** | **Ashamed** | **Guilty** | **Lonely** | **Sad** | **Restless** | **Tired** | **Hopeless** |
| --- | --- | --- | --- | --- | --- | --- | --- | --- | --- | --- | --- | --- | --- | --- | --- | --- | --- | --- | --- | --- | --- | --- |
| **Determined** | 0.66 | -- | -- | -- | -- | -- | -- | -- | -- | -- | -- | -- | -- | -- | -- | -- | -- | -- | -- | -- | -- | -- |
| **Enthusiastic** | 0.74 | 0.70 | -- | -- | -- | -- | -- | -- | -- | -- | -- | -- | -- | -- | -- | -- | -- | -- | -- | -- | -- | -- |
| **Proud** | 0.61 | 0.59 | 0.65 | -- | -- | -- | -- | -- | -- | -- | -- | -- | -- | -- | -- | -- | -- | -- | -- | -- | -- | -- |
| **Happy** | 0.72 | 0.62 | 0.73 | 0.80 | -- | -- | -- | -- | -- | -- | -- | -- | -- | -- | -- | -- | -- | -- | -- | -- | -- | -- |
| **Joyful** | 0.69 | 0.63 | 0.71 | 0.79 | 0.89 | -- | -- | -- | -- | -- | -- | -- | -- | -- | -- | -- | -- | -- | -- | -- | -- | -- |
| **Attentive** | 0.59 | 0.66 | 0.69 | 0.60 | 0.63 | 0.70 | -- | -- | -- | -- | -- | -- | -- | -- | -- | -- | -- | -- | -- | -- | -- | -- |
| **Calm** | 0.69 | 0.62 | 0.63 | 0.58 | 0.74 | 0.74 | 0.63 | -- | -- | -- | -- | -- | -- | -- | -- | -- | -- | -- | -- | -- | -- | -- |
| **Grateful** | 0.49 | 0.69 | 0.58 | 0.61 | 0.66 | 0.65 | 0.60 | 0.57 | -- | -- | -- | -- | -- | -- | -- | -- | -- | -- | -- | -- | -- | -- |
| **Relaxed** | 0.67 | 0.57 | 0.63 | 0.63 | 0.78 | 0.73 | 0.62 | 0.78 | 0.57 | -- | -- | -- | -- | -- | -- | -- | -- | -- | -- | -- | -- | -- |
| **Angry** | -0.30 | -0.35 | -0.32 | -0.30 | -0.40 | -0.43 | -0.35 | -0.45 | -0.43 | -0.33 | -- | -- | -- | -- | -- | -- | -- | -- | -- | -- | -- | -- |
| **Disgusted** | -0.35 | -0.37 | -0.35 | -0.38 | -0.47 | -0.43 | -0.39 | -0.40 | -0.43 | -0.44 | 0.80 | -- | -- | -- | -- | -- | -- | -- | -- | -- | -- | -- |
| **Irritable** | -0.36 | -0.37 | -0.44 | -0.35 | -0.46 | -0.49 | -0.40 | -0.45 | -0.39 | -0.43 | 0.69 | 0.67 | -- | -- | -- | -- | -- | -- | -- | -- | -- | -- |
| **Nervous** | -0.35 | -0.34 | -0.38 | -0.35 | -0.45 | -0.41 | -0.39 | -0.46 | -0.38 | -0.49 | 0.60 | 0.73 | 0.74 | -- | -- | -- | -- | -- | -- | -- | -- | -- |
| **Scared** | -0.36 | -0.38 | -0.34 | -0.35 | -0.44 | -0.41 | -0.36 | -0.45 | -0.40 | -0.45 | 0.64 | 0.68 | 0.67 | 0.78 | -- | -- | -- | -- | -- | -- | -- | -- |
| **Ashamed** | -0.24 | -0.34 | -0.26 | -0.27 | -0.35 | -0.30 | -0.23 | -0.33 | -0.34 | -0.27 | 0.78 | 0.82 | 0.61 | 0.63 | 0.69 | -- | -- | -- | -- | -- | -- | -- |
| **Guilty** | -0.38 | -0.35 | -0.37 | -0.41 | -0.47 | -0.49 | -0.40 | -0.46 | -0.43 | -0.42 | 0.73 | 0.81 | 0.76 | 0.76 | 0.79 | 0.75 | -- | -- | -- | -- | -- | -- |
| **Lonely** | -0.34 | -0.26 | -0.30 | -0.38 | -0.47 | -0.44 | -0.32 | -0.39 | -0.38 | -0.39 | 0.49 | 0.54 | 0.58 | 0.57 | 0.60 | 0.57 | 0.58 | -- | -- | -- | -- | -- |
| **Sad** | -0.43 | -0.42 | -0.49 | -0.47 | -0.57 | -0.56 | -0.41 | -0.47 | -0.51 | -0.53 | 0.71 | 0.76 | 0.76 | 0.79 | 0.80 | 0.66 | 0.78 | 0.69 | -- | -- | -- | -- |
| **Restless** | -0.31 | -0.28 | -0.25 | -0.29 | -0.41 | -0.38 | -0.30 | -0.36 | -0.34 | -0.41 | 0.48 | 0.50 | 0.61 | 0.56 | 0.58 | 0.50 | 0.52 | 0.62 | 0.60 | -- | -- | -- |
| **Tired** | -0.55 | -0.26 | -0.40 | -0.33 | -0.50 | -0.49 | -0.38 | -0.44 | -0.28 | -0.51 | 0.46 | 0.48 | 0.55 | 0.54 | 0.52 | 0.39 | 0.53 | 0.42 | 0.57 | 0.61 | -- | -- |
| **Hopeless** | -0.29 | -0.37 | -0.32 | -0.34 | -0.46 | -0.43 | -0.32 | -0.40 | -0.45 | -0.43 | 0.60 | 0.65 | 0.67 | 0.68 | 0.84 | 0.67 | 0.69 | 0.67 | 0.80 | 0.66 | 0.44 | -- |
| **Bored** | -0.41 | -0.32 | -0.42 | -0.41 | -0.50 | -0.45 | -0.38 | -0.34 | -0.37 | -0.47 | 0.48 | 0.57 | 0.61 | 0.61 | 0.64 | 0.52 | 0.59 | 0.71 | 0.72 | 0.68 | 0.61 | 0.62 |

*Note.* All associations are significant at p<.001.

Supplementary Table 2. Within-person correlations among all affective states.

|  | **Active** | **Determined** | **Enthusiastic** | **Proud** | **Happy** | **Joyful** | **Attentive** | **Calm** | **Grateful** | **Relaxed** | **Angry** | **Disgusted** | **Irritable** | **Nervous** | **Scared** | **Ashamed** | **Guilty** | **Lonely** | **Sad** | **Restless** | **Tired** | **Hopeless** |
| --- | --- | --- | --- | --- | --- | --- | --- | --- | --- | --- | --- | --- | --- | --- | --- | --- | --- | --- | --- | --- | --- | --- |
| **Determined** | 0.28 | -- | -- | -- | -- | -- | -- | -- | -- | -- | -- | -- | -- | -- | -- | -- | -- | -- | -- | -- | -- | -- |
| **Enthusiastic** | 0.30 | 0.35 | -- | -- | -- | -- | -- | -- | -- | -- | -- | -- | -- | -- | -- | -- | -- | -- | -- | -- | -- | -- |
| **Proud** | 0.21 | 0.28 | 0.34 | -- | -- | -- | -- | -- | -- | -- | -- | -- | -- | -- | -- | -- | -- | -- | -- | -- | -- | -- |
| **Happy** | 0.25 | 0.27 | 0.42 | 0.39 | -- | -- | -- | -- | -- | -- | -- | -- | -- | -- | -- | -- | -- | -- | -- | -- | -- | -- |
| **Joyful** | 0.25 | 0.25 | 0.38 | 0.34 | 0.45 | -- | -- | -- | -- | -- | -- | -- | -- | -- | -- | -- | -- | -- | -- | -- | -- | -- |
| **Attentive** | 0.27 | 0.29 | 0.34 | 0.28 | 0.28 | 0.38 | -- | -- | -- | -- | -- | -- | -- | -- | -- | -- | -- | -- | -- | -- | -- | -- |
| **Calm** | 0.19 | 0.27 | 0.34 | 0.27 | 0.40 | 0.30 | 0.17 | -- | -- | -- | -- | -- | -- | -- | -- | -- | -- | -- | -- | -- | -- | -- |
| **Grateful** | 0.17 | 0.29 | 0.30 | 0.28 | 0.30 | 0.25 | 0.22 | 0.24 | -- | -- | -- | -- | -- | -- | -- | -- | -- | -- | -- | -- | -- | -- |
| **Relaxed** | 0.11 | 0.21 | 0.30 | 0.24 | 0.39 | 0.39 | 0.31 | 0.40 | 0.21 | -- | -- | -- | -- | -- | -- | -- | -- | -- | -- | -- | -- | -- |
| **Angry** | -0.09 | -0.15 | -0.23 | -0.19 | -0.32 | -0.25 | -0.15 | -0.33 | -0.18 | -0.29 | -- | -- | -- | -- | -- | -- | -- | -- | -- | -- | -- | -- |
| **Disgusted** | -0.12 | -0.13 | -0.19 | -0.20 | -0.30 | -0.25 | -0.15 | -0.28 | -0.18 | -0.27 | 0.45 | -- | -- | -- | -- | -- | -- | -- | -- | -- | -- | -- |
| **Irritable** | -0.15 | -0.15 | -0.24 | -0.19 | -0.34 | -0.30 | -0.18 | -0.30 | -0.20 | -0.34 | 0.42 | 0.43 | -- | -- | -- | -- | -- | -- | -- | -- | -- | -- |
| **Nervous** | -0.05 | -0.11 | -0.14 | -0.11 | -0.21 | -0.17 | -0.09 | -0.20 | -0.16 | -0.23 | 0.25 | 0.28 | 0.36 | -- | -- | -- | -- | -- | -- | -- | -- | -- |
| **Scared** | -0.08 | -0.12 | -0.14 | -0.17 | -0.20 | -0.17 | -0.12 | -0.19 | -0.15 | -0.20 | 0.29 | 0.32 | 0.25 | 0.36 | -- | -- | -- | -- | -- | -- | -- | -- |
| **Ashamed** | -0.07 | -0.13 | -0.14 | -0.19 | -0.21 | -0.18 | -0.12 | -0.18 | -0.16 | -0.17 | 0.36 | 0.36 | 0.26 | 0.24 | 0.29 | -- | -- | -- | -- | -- | -- | -- |
| **Guilty** | -0.09 | -0.16 | -0.15 | -0.19 | -0.19 | -0.20 | -0.11 | -0.18 | -0.16 | -0.16 | 0.26 | 0.34 | 0.27 | 0.30 | 0.32 | 0.43 | -- | -- | -- | -- | -- | -- |
| **Lonely** | -0.13 | -0.12 | -0.15 | -0.15 | -0.19 | -0.17 | -0.13 | -0.14 | -0.13 | -0.15 | 0.22 | 0.23 | 0.25 | 0.28 | 0.28 | 0.24 | 0.25 | -- | -- | -- | -- | -- |
| **Sad** | -0.12 | -0.17 | -0.22 | -0.22 | -0.29 | -0.26 | -0.17 | -0.24 | -0.20 | -0.27 | 0.35 | 0.37 | 0.36 | 0.40 | 0.44 | 0.29 | 0.32 | 0.33 | -- | -- | -- | -- |
| **Restless** | -0.15 | -0.14 | -0.18 | -0.16 | -0.23 | -0.23 | -0.14 | -0.17 | -0.11 | -0.26 | 0.25 | 0.27 | 0.34 | 0.34 | 0.29 | 0.20 | 0.24 | 0.25 | 0.36 | -- | -- | -- |
| **Tired** | -0.27 | -0.13 | -0.17 | -0.11 | -0.19 | -0.22 | -0.17 | -0.11 | -0.09 | -0.17 | 0.16 | 0.19 | 0.24 | 0.22 | 0.23 | 0.15 | 0.18 | 0.18 | 0.25 | 0.39 | -- | -- |
| **Hopeless** | -0.11 | -0.12 | -0.15 | -0.16 | -0.19 | -0.18 | -0.12 | -0.17 | -0.19 | -0.17 | 0.27 | 0.31 | 0.30 | 0.30 | 0.44 | 0.24 | 0.25 | 0.25 | 0.43 | 0.29 | 0.20 | -- |
| **Bored** | -0.15 | -0.13 | -0.12 | -0.16 | -0.18 | -0.20 | -0.14 | -0.11 | -0.13 | -0.14 | 0.16 | 0.23 | 0.24 | 0.24 | 0.37 | 0.18 | 0.19 | 0.31 | 0.27 | 0.31 | 0.27 | 0.31 |

*Note.* All associations are significant at p<.001.
